# Geographic range, distribution patterns and interactions of Monogenea Van Beneden 1858, with species of native host freshwater fishes from Brazil

**DOI:** 10.1590/S1984-29612022048

**Published:** 2022-08-22

**Authors:** Marcos Tavares-Dias, Luís Mauricio Abdon Silva, Marcos Sidney Brito Oliveira

**Affiliations:** 1 Embrapa Amapá, Macapá, AP, Brasil; 2 Programa de Pós-Graduação em Biodiversidade Tropical – PPGBio, Universidade Federal do Amapá – UNIFAP, Macapá, AP, Brasil; 3 Núcleo de Pesquisas Aquáticas – NUPAq, Instituto de Pesquisas Científicas e Tecnológicas do Estado do Amapá – IEPA, Macapá, AP, Brasil

**Keywords:** Hosts, freshwater fish, infection, monogeneans, parasites, Hospedeiros, peixes de água doce, infecção, monogenéticos, parasitos

## Abstract

This paper investigated information on monogenean species using 312 scientific papers, to search for infection and geographic distribution patterns in native freshwater fish from Brazil. We used 1,698 samples of 296 fish species of 28 families distributed into Characiformes, Siluriformes, Cichliformes, Gymnotiformes, Perciformes, Mugiliformes, Osteoglossiformes and Clupeiformes, in addition to four hybrid fish. Among the hosts of the different orders and families, the greatest numbers of parasite-host associations were found for species of the families Serrasalmidae, Characidae, Loricariidae, Curimatidae and Anostomidae. The 578 species of monogeneans used in parasite-host interactions were distributed in 86 genera of six five families (Dactylogyridae, Gyrodactylidae, Diplectanidae, Microcotylidae, Ancylodiscoididae and Ancyrocephalidae), but with great predominance of Dactylogyridae species. There was variation in prevalence, intensity and abundance levels of monogeneans species among host fish species, as well as in infection sites that occurred predominantly in external organs. Positive correlations of prevalence, intensity and abundance with body length of hosts were observed. There was geographic distribution pattern of monogeneans limited mostly to two hydrographic basins those being the Amazon River and Paraná River. Just approximately 6% of potential monogeneans have been explored thus far, showing a clear need for further studies on this interesting group of parasites.

## Resumo

Este estudo investigou informações sobre espécies de monogenéticos, usando 312 artigos científicos para buscar padrões de infecção e distribuição geográfica em peixes nativos de água doce do Brasil. Foram utilizadas 1.698 amostras de 296 espécies de peixes de 28 famílias, distribuídas em Characiformes, Siluriformes, Cichliformes, Gymnotiformes, Perciformes, Mugiliformes, Osteoglossiformes e Clupeiformes, além de quatro peixes híbridos. Entre os hospedeiros das diferentes ordens e famílias, os maiores números de associações parasito-hospedeiro foram encontrados para espécies das famílias Serrasalmidae, Characidae, Loricariidae, Curimatidae e Anostomidae. As 578 espécies de monogenéticos, utilizadas nas interações parasito-hospedeiro, foram distribuídas em 86 gêneros de seis famílias (Dactylogyridae, Gyrodactylidae, Diplectanidae, Microcotylidae, Ancylodiscoididae e Ancyrocephalidae), mas com grande predominância de espécie de Dactylogyridae. Houve variação nos níveis de prevalência, intensidade e abundância das espécies de monogenéticos entre as espécies de peixes hospedeiros, bem como nos locais de infecção que ocorreram predominantemente em órgãos externos. Correlações positivas de prevalência, intensidade e abundância com o comprimento corporal dos hospedeiros foram observadas. Houve padrão de distribuição geográfica dos monogenéticos limitados principalmente a duas bacias hidrográficas, sendo elas o Rio Amazonas e Rio Paraná. Apenas aproximadamente 6% dos potenciais monogenéticos são conhecidos até agora, mostrando uma clara necessidade de mais estudos sobre esse interessante grupo de parasitos.

## Introduction

Monogenea Van Beneden 1858, a class parasitic Platyhelminthes, are highly diverse in marine, brackish and freshwater fish, sharks, rays, and even chelonians, amphibians and crustaceans. This group of obligate parasites has simple and direct life cycle that allows them to rapidly multiply in lentic and eutrophized environments such as aquaculture, where these parasites often have a harmful impact on their hosts’ health (Cohen et al., 2013; Takemoto et al., 2013). The oncomiracidium is the larval stage of monogeneans and has the task of locating, attaching and establishing itself on the hosts (Cohen et al., 2013; Takemoto et al., 2013); hence there are limitations on their ability to disperse through environments. Most monogeneans are ectoparasites that move about freely on the fish’s body surface feed on mucus and epithelial cells of the skin and gills, and a few adult monogeneans will remain permanently attached to a single site on the host. However, a few species of monogeneans are endoparasites inhabiting digestive tract, heart, kidney, blood vessels or urinary bladder of host fish (Cribb et al., 2002; Cohen et al., 2013; Hoai, 2020; Kuchta et al., 2020). In addition, better survival of a monogenean species in a certain microhabitat due to decreased exposure to adverse abiotic or biotic factors, including predation by fish or other animals, could direct habitat selection (Buchmann & Lindenstrøm, 2002; Gobbin et al., 2021).

Monogenea is a species-rich class of helminths and is strictly parasitic and diverse with respect to their morphology and ecology of host fishes (Braga et al., 2014). In Brazil, until 1965, only *Fredericianella ovicola* (Brandes, 1894) and *Capsala laevis* (Verril, 1874) infecting fish had been described (Cohen et al., 2013). In 1989, Kohn & Santos (1989) listed monogenean species in fishes from Brazil. Later, new lists of host fishes from South America, including Brazil, infected by monogeneans species have been carried out by Kohn & Cohen (1998), Cohen & Kohn (2008) and Cohen et al. (2013). Takemoto et al. (2013) also listed the freshwater monogeneans species in fishes from Brazil. Currently, studies reported that among the helminth parasites of teleost fishes, monogeneans represent a large and specious group with an estimated 552 known species (Luque et al., 2017). However, this estimate can be even higher, since the fauna composition of monogeneans can vary in a single fish species, according to the geographic distribution and the specific site in the host (Boeger & Kritsky, 1988). However, these studies represent only a part of knowledge on fish species and monogeneans in hosts fish populations from Brazil.

Occurrences of diseases caused by monogeneans in farmed fish have increased as the number and size of fish farms have grown. This increase is due to high density of fish and parasites in ponds resulting in enhanced opportunity for infestations and epizooties. Monogeneans usually are well accommodated to their wild host fish causing few easily detectable effects. Contrarily, they frequently cause severe epizooties in farmed fish populations (Grano-Maldonado et al., 2018; Hoai, 2020), which do not seem occur in wild fish populations. The presence of monogeneans in the gills of the fish can cause cellular hyperplasia, excessive production of mucus and fusion of filaments of the branchial lamellae, resulting in breathing difficulties and mortality (Kaur & Shrivastav, 2014; Igeh & Avenant-Oldewage, 2020; Tavares-Dias et al., 2021), due to difficulty of obtaining oxygen in highly infected fish. In the integument of fish, monogeneans usually cause fewer injuries, but can serve as a gateway for the installation of other infections caused by fungus and bacteria. In such cases, the short, direct, one-host life cycles of monogeneans enable them to reach epizootic levels very quickly when hosts and parasites are confined closely together (Hoai, 2020). Also, the infection levels of many monogenean species responded to changes in the physical and chemical water parameters of their environment (Lacerda et al., 2018; Cavalcanti et al., 2020).

Monogenea is the group of parasites with more high specificity of hosts when compared to other groups of helminths, i.e., each monogenean species infects only one or few host species or a few host families (Whittington et al., 2000; Poulin, 2002; Buchmann & Lindenstrøm, 2002; Braga et al., 2014; Řehulková et al., 2021). This high host specificity showed by many monogenean species makes it easier to search for a link between the ecological characteristics of the hosts and the diversity of their parasites (Poulin, 2002). Mechanisms responsible for host specificity are one of the main issues in parasitology that has been studied for decades. As far as monogeneans parasitizing teleost fish is concerned, this question is even more relevant to pose (Buchmann & Lindenstrøm, 2002; Řehulková et al., 2021).

Studies on the parasitic monogenean diversity are significant from many perspectives. First, these parasites, similar to any other organism, form an integral part of global biodiversity and, therefore, they provide important information on the structure and functioning of ecosystems. Second, these parasites diversity can provide information on host phylogeny and ecology. Many studies on fish parasites groups from Brazil have been carried out, showing a variation of for helminth species, including monogeneans. However, studies on the geographical distribution and distribution patterns of monogeneans occurrence in host fishes of have been not carried out. Thus, the present study aimed to characterize the geographic range, distribution patterns and interactions of monogenean species associated with Brazilian freshwater native host fishes of different basins throughout the country. This study is of great interest because the knowledge of the monogeneans patterns for fish in Brazil may contribute to global estimates of the distribution of these parasites in fish.

## Materials and Methods

A review on the Monogenea species in freshwater native teleost fish of Brazil was performed by searching databases (Scielo, ISI, Scopus, Science Direct, Zoological Records, CAB Abstracts, Lilacs, Capes periodical and Google Scholar), and available data in 312 scientific papers that were systematized and used. Terms used in searching were monogenea, fish and Brazil. All articles on the monogenean associated to fishes were used. A dataset of monogenean species parasitizing freshwater fish populations in Brazil was compiled using taxonomic descriptions of species and surveys on the occurrences of these parasites published between 1979 and 2022. These data comprised surveys on monogenean species of native fish in rivers, lakes, lagoons and reservoirs distributed throughout Brazil, except for 197 samples of hosts that were of farmed fish. These surveys were chosen because they represent the various aquatic ecosystems found in Brazil and may aid in revealing the distribution patterns and interactions of monogenean species with host fishes. In addition, comparisons were performed between the parasite samples of wild fish and aquaculture fish. We used sample data of prevalence, intensity and abundance obtained from published research.

The taxonomy of fish was obtained from Froese & Pauly (2022) for each host species, and the sampling unit was considered as the number of individuals parasitized by a monogenean species at a certain location and time. Some of the information used in samples included data on more than one host species. The data were organized in a data frame (extension “.txt”) with a list of the following variables: (i) parasite species, (ii) infection site, (iii) mean prevalence, (iv) mean intensity and (v) mean abundance; along with categorical factors such as: (i) host fish species, (ii) locality of sample collection, (iii) family and order of host and (iv) mean length and weight of the hosts.

In order to present the geographic distribution of monogenean species for the different Brazilian hydrographic basins (Amazon River, Tocantins River, São Francisco River, Paraná River, Uruguay River, East Atlantic, North Atlantic and Southeast Atlantic), we compiled the coordinates of the parasite collection sites in the articles in the database. For articles that do not report the geographic coordinates of the host fish collection, we plot the reference point provided in the article. The plotting of coordinates was done using Google Earth software and the generated database was exported to Quantum-Gis (QGIS) for making the map.

## Data Analysis

The ecological terms used were those recommended by Rohde et al. (1995) and Bush et al. (1997). The prevalence, abundance and intensity data on the parasites were tested for normal distribution and homoscedasticity of variances, and these infection parameters did not present a normal distribution (Zar, 2010).

Spearman’s correlation coefficient (*rs*) between monogeneans prevalence, intensity, abundance and length of host fish was used to ascertain whether larger fish tended to have higher prevalence, intensity and abundance of parasites transformed in log scale. Firstly, the normality of the data was evaluated through the Shapiro-Wilk test, since the data did not present normal distribution, even after logarithmic transformation and obtaining the arcsine square root, the Spearman correlation test was then used (Zar, 2010). In order to define interaction patterns, networks were drawn with plot web function in the package bipartite. These analyses and factors were analyzed with the aim of producing a classification according to groups of parasites, using R with the “package bipartite” (Dormann et al., 2008; Dormann, 2011; R Core Team, 2017).

To determine the monogeneans-host relationships at species level, a bipartite package (Dormann et al., 2008) was used to construct a bipartite network, in addition to calculating network-level indices, such as the c-score, number of compartments and specificity index of the species (SSI) (Dormann, 2011). The c-score index measures the co-occurrence rate of species in the network and is an indicator of the degree of specificity of the species that compose it, with values ranging from 0 (high co-occurrence) to 1 (low co-occurrence). Compartments are independent groups of ectoparasites and hosts within the network and are indicators of patterns of specificity. The SSI measures the level of species specificity of parasites, ranging from 0 (low specificity) to 1 (high specificity). Range is the number of fish species with which a species of ectoparasites interacts. Finally, the strength of species is the sum of the participation proportions of a species in all interactions within the network. The volume of connecting bars and lines represents the proportion of interactions performed by each species, and between species, respectively. These analyzes were performed using the R software package (R Core Team, 2017).

## Results

Our search resulted in a total of 1,698 samples of monogeneans that were found to parasitize 296 fish species (including 4 hybrids) of 28 families and 8 orders. The species richness of monogeneans and families of host fishes are presented in Table 1, which show a higher number of occurrences on Serrasalmidae species, principally due to their distribution in basins from Amazonas River and Paraná River.

**Table 1:** Richness of Monogenea species (n= 578) by taxonomic groups in 296 freshwater host fish from Brazil.

**Host order**	**Host family**	**Host species number**	**Parasite species richness**
Characiformes	Acestrorhynchidae	3	5
	Anostomidae	22	34
	Bryconidae	6	23
	Characidae	46	50
	Crenuchidae	3	5
	Curimatidae	7	13
	Cynodontidae	2	6
	Erythrinidae	5	31
	Parodontidae	3	4
	Prochilodontidae	4	23
	Serrasalmidae	25	97
	Triportheidae	6	25
	Hemiodontidae	1	1
Siluriformes	Callichthyidae	7	10
	Doradidae	7	18
	Heptapteridae	3	10
	Auchenipteridae	6	16
	Pimelodidae	19	58
	Loricariidae	41	72
	Ariidae	1	4
Perciformes	Centropomidae	1	6
	Sciaenidae	6	17
Cichliformes	Cichlidae	37	46
Gymnotiformes	Gymnotidae	2	4
Mugiliformes	Mugilidae	2	5
Osteoglossiformes	Osteoglossidae	1	6
	Arapaimidae	1	3
Clupeiformes	Pristigasteridae	1	1
Hibrid *Colossoma macropomum* x *Piaractus mesopotamicus*		1	2
Hybrid *Colossoma macropomum* x *Piaractus brachypomus*		1	5
Hybrid *Piaractus mesopotamicus* x *Piaractus brachypomus*		1	5
Hybrid *Pseudoplatystoma reticulata x Pseudoplatystoma corruscans*		1	5

This study was performed with a total of 578 monogenean species and most of them were in wild fish (91.8%). In samples, five families of monogeneans: Dactylogyridae (n = 1,509), Gyrodactylidae (n = 152), Diplectanidae (n = 24), Microcotylidae (n = 8), Ancylodiscoididae (n = 4) and Ancyrocephalidae (n = 1) were found. A total of 492 species of Dactylogyridae, 60 species of Gyrodactylidae, 17 species of Diplectanidae, 4 species of Microcotylidae, 4 species of Ancylodiscoididae and 1 species of Ancyrocephalidae was found. However, there was great predominance of Dactylogyridae.

**Figure 1:**
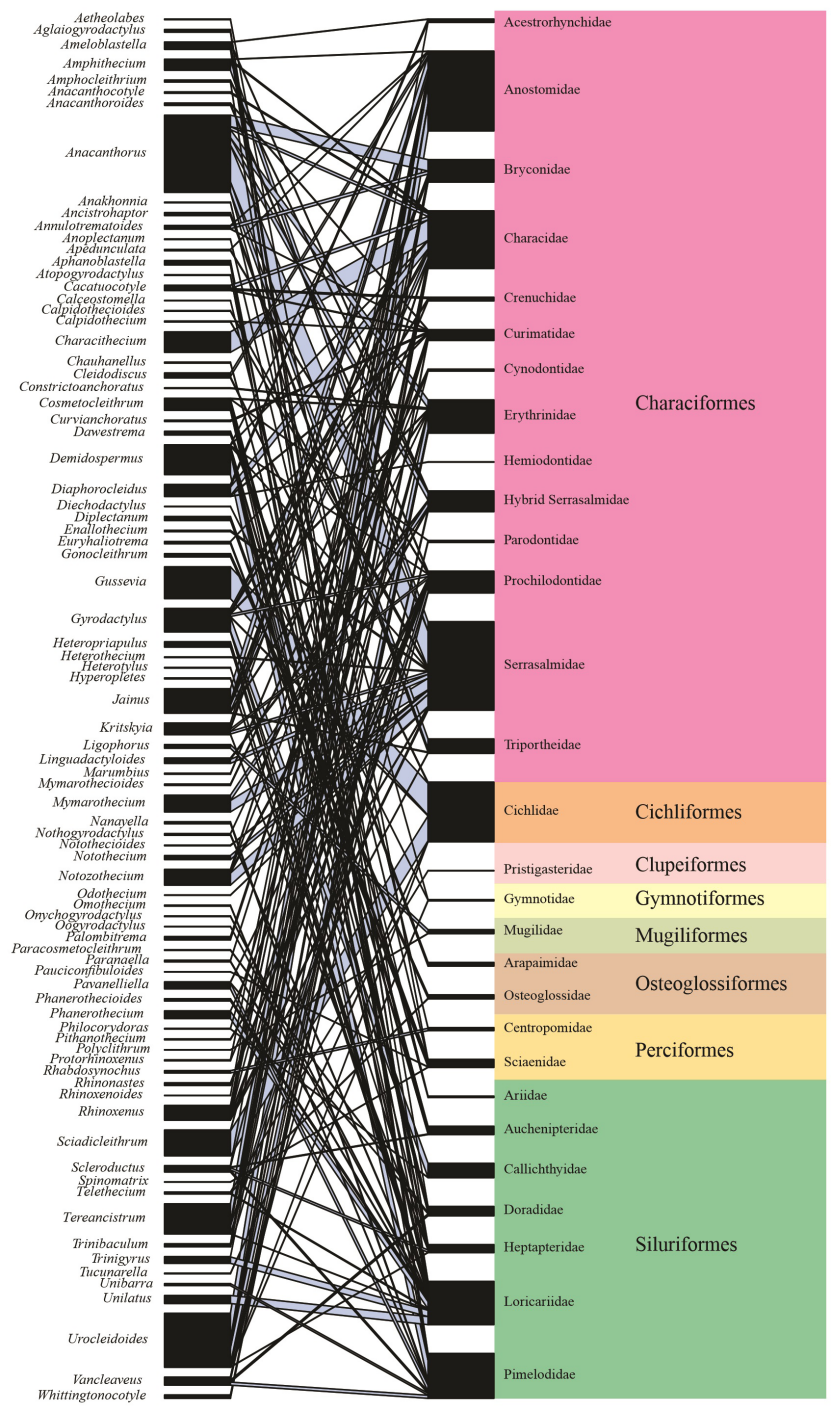
Network of interactions between species of host fish (n = 296) from Brazil and species of Monogenea.

In the interaction network relating Monogenea genera with host fish families, the rate of co-occurrence of these parasites was low at the network level (c-score = 0.833), indicating that the parasites do not share the same families of hosts, because these have some specificity by families of hosts. The interaction between monogenean genera and host families was mostly recorded in only one host family in the network (Figure 1). In general, all Monogenea genera were considered specialists for host families, with a range of specificity levels ranging from high to moderate (Table 2). The genera of Monogenea with the highest number of host families were the *Demidospermus* Suriano, 1983 (n = 9), *Urocleidoides* Mizelle & Price, 1964 (n = 9), *Gyrodactylus* von Nordmann, 1832 (n = 8) and *Anacanthorus* Mizelle & Price, 1965 (n = 7).

**Table 2:** Specificity indices for genera of Monogenea parasites of freshwater fish from Brazil.

**Monogenea**	**Degree**	**Normalised degree**	**Species strength**	**Species specificity index (SSI)**	**Level of specificity**	**Proportional generality**
*Aetheolabes*	1	0.034	0.083	1.000	High	0.065
*Aglaiogyrodactylus*	1	0.034	0.062	1.000	High	0.065
*Ameloblastella*	5	0.172	0.345	0.733	High	0.164
*Amphithecium*	3	0.103	0.133	0.851	High	0.107
*Amphocleithrium*	2	0.068	0.070	0.693	High	0.129
*Anacanthocotyle*	1	0.034	0.023	1.000	High	0.065
*Anacanthoroides*	2	0.068	0.139	0.759	High	0.118
*Anacanthorus*	7	0.241	2.521	0.513	Moderate	0.294
*Anakhonnia*	1	0.034	0.222	1.000	High	0.064
*Ancistrohaptor*	1	0.034	0.209	1.000	High	0.064
*Annulotrematoides*	3	0.103	0.169	0.821	High	0.118
*Anoplectanum*	1	0.034	0.083	1.000	High	0.064
*Apedunculata*	2	0.068	0.050	0.781	High	0.114
*Aphanoblastella*	2	0.068	0.473	0.854	High	0.099
*Atopogyrodactylus*	1	0.034	0.015	1.000	High	0.064
*Cacatuocotyle*	3	0.103	0.484	0.670	High	0.064
*Calceostomella*	1	0.034	0.250	1.000	High	0.159
*Calpidothecioides*	1	0.034	0.007	1.000	High	0.064
*Calpidothecium*	2	0.068	0.038	0.771	High	0.122
*Characithecium*	1	0.034	0.354	1.000	High	0.064
*Chauhanellus*	1	0.034	0.750	1.000	High	0.064
*Cleidodiscus*	2	0.068	0.068	0.770	High	0.116
*Constrictoanchoratus*	1	0.034	0.020	1.000	High	0.064
*Cosmetocleithrum*	5	0.172	1.401	0.616	Moderate	0.191
*Curvianchoratus*	1	0.034	0.125	1.000	High	0.064
*Dawestrema*	1	0.034	1.000	1.000	High	0.064
*Demidospermus*	9	0.310	0.989	0.733	High	0.175
*Diaphorocleidus*	4	0.137	2.007	0.720	High	0.151
*Diechodactylus*	1	0.034	0.250	1.000	High	0.064
*Diplectanum*	1	0.034	0.500	1.000	High	0.064
*Enallothecium*	1	0.034	0.015	1.000	High	0.064
*Euryhaliotrema*	2	0.068	0.281	0.863	High	0.097
*Gonocleithrum*	1	0.034	0.833	1.000	High	0.064
*Gussevia*	1	0.034	0.533	1.000	High	0.064
*Gyrodactylus*	8	0.275	1.597	0.611	Moderate	0.247
*Heteropriapulus*	1	0.034	0.124	1.000	High	0.064
*Heterothecium*	1	0.034	0.007	1.000	High	0.064
*Heterotylus*	1	0.034	0.015	1.000	High	0.064
*Hyperopletes*	1	0.034	0.023	1.000	High	0.064
*Jainus*	5	0.172	0.511	0.584	Moderate	0.216
*Kritskyia*	5	0.172	0.307	0.555	Moderate	0.232
*Ligophorus*	1	0.034	0.916	1.000	High	0.064
*Linguadactyloides*	2	0.068	0.109	0.782	High	0.114
*Marumbius*	1	0.034	0.272	1.000	High	0.064
*Mymarothecioides*	1	0.034	0.666	1.000	High	0.064
*Mymarothecium*	2	0.068	0.386	0.741	High	0.121
*Nanayella*	1	0.034	0.044	1.000	High	0.064
*Nothogyrodactylus*	2	0.068	0.041	0.817	High	0.107
*Notothecioides*	1	0.034	0.007	1.000	High	0.064
*Notothecium*	1	0.034	0.045	1.000	High	0.064
*Notozothecium*	4	0.137	0.423	0.772	High	0.138
*Odothecium*	1	0.034	0.003	1.000	High	0.064
*Omothecium*	1	0.034	0.022	1.000	High	0.064
*Onychogyrodactylus*	1	0.034	0.015	1.000	High	0.064
*Oogyrodactylus*	1	0.034	0.007	1.000	High	0.064
*Palombitrema*	1	0.034	0.312	1.000	High	0.064
*Paracosmetocleithrum*	1	0.034	0.071	1.000	High	0.064
*Paranaella*	2	0.068	0.085	0.709	High	0.127
*Pauciconfibuloides*	1	0.034	0.041	1.000	High	0.064
*Pavanelliella*	3	0.103	0.200	0.858	High	0.107
*Phanerothecioides*	1	0.034	0.054	1.000	High	0.064
*Phanerothecium*	2	0.068	0.185	0.955	High	0.077
*Philocorydoras*	2	0.068	0.054	0.693	High	0.129
*Pithanothecium*	1	0.034	0.011	1.000	High	0.064
*Polyclithrum*	1	0.034	0.083	1.000	High	0.064
*Protorhinoxenus*	2	0.068	0.023	0.734	High	0.122
*Rhabdosynochus*	1	0.034	0.777	1.000	High	0.060
*Rhinonastes*	1	0.034	0.123	1.000	High	0.064
*Rhinoxenoides*	1	0.034	0.100	1.000	High	0.064
*Rhinoxenus*	6	0.206	0.492	0.572	Moderate	0.247
*Sciadicleithrum*	2	0.068	0.446	0.960	High	0.076
*Scleroductus*	5	0.172	0.445	0.492	Moderate	0.275
*Spinomatrix*	1	0.034	0.041	1.000	High	0.064
*Telethecium*	3	0.103	1.189	0.605	Moderate	0.178
*Tereancistrum*	5	0.172	0.696	0.679	High	0.171
*Trinibaculum*	4	0.137	0.075	0.508	Moderate	0.240
*Trinigyrus*	2	0.068	0.153	0.949	High	0.079
*Tucunarella*	1	0.034	0.011	1.000	High	0.064
*Unibarra*	1	0.034	0.044	1.000	High	0.064
*Unilatus*	1	0.034	0.186	1.000	High	0.064
*Urocleidoides*	9	0.310	2.504	0.566	Moderate	0.246
*Vancleaveus*	4	0.137	0.420	0.539	Moderate	0.218
*Whittingtonocotyle*	1	0.034	0.101	1.000	High	0.064

In the samples, species of *Gyrodactylus* von Nordmann, 1832 occurred in 90.0% of Callichthyidae hosts, while the other Siluriformes were infected mostly by Dactylogyridae species. Species of *Anacanthorus* infected predominantly Bryconidae, Serrasalmidae and Erythrinidae hosts. Species of *Gussevia* Kohn & Paperna 1964 infected Cichlidae hosts, while species of *Demidospermus* Suriano 1983 occurred mainly in Pimelodidade hosts. Species of *Jainus* Mizelle, Kritsky & Crane 1967 occurred in Characidae, Bryconidae and Triportheidae, but were more frequent in Anostomidae hosts. Species of *Sciadicleithrum* Kritsky, Thatcher & Boeger 1989 infected predominantly Cichlidae hosts, however, species of *Urocleidoides* occurred in Anostomidae and Erythrinidae hosts, as well as in few Characidae and Curimatidae hosts. Species of *Characithecium* Mendoza-Franco, Reina, & Torchin, 2009 infected predominantly Characidae. Species of *Notozothecium* Boeger & Kritsky 1988 and *Mymarothecium* Kritsky, Boeger & Jégu 1996 infected predominantly Serrasalmidae. Species of *Cosmetocleithrum* Kritsky, Thatcher & Boeger, 1986 infected predominantly Doradidae and Auchenipteridae. Species of *Diaphorocleidus* Jogunoori, Kritsky & Venkatanarasaiah 2004, infected predominantly Characidae, Acestrorhynchidae, but also occurred in Hemiodontidae and Curimatidae (Figure 1).

**Figure 2:**
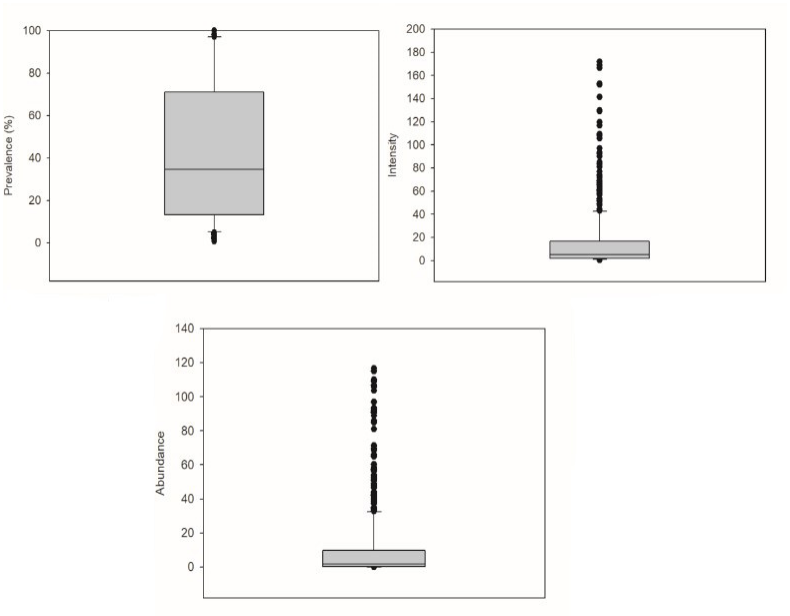
Quantitative descriptors of infection for Monogenea species in samples of freshwater fish from Brazil (Box plots represent medians, percentile ranges (25-75%), minimum-maximum and outlier values).

In the host fish analyzed, data of prevalence, intensity and abundance of monogenean species are shown in Table 3 and Figure 2. The prevalence was equal to 100% in 17.5% of host species and higher intensity and abundance were in farmed fish species.

**Table 3:** Parameters of infection by monogeneans in species of freshwater fish in Brazil.

**Parameters**	**N**	**Median**	**25^th^ percentile**	**75^th^ percentile**	**Minimum**	**Maximum**
Prevalence (%)	958	34.6	13.3	71.0	0.7	100
Intensity	696	5.2	2.1	16.5	0.3	171.7
Abundance	619	1.8	0.3	9.7	0.006	116.7

Infection sites of monogenean species occurred in both internal and external organs such as gills and nostrils (0.9%), gills, nostrils and tegument (1.3%), gills and tegument (3.2%), gills (80.8%), tegument (8%), tegument and nostrils (0.1%), head pores (0.07%), nostrils (3.9%), urinary bladder (0.5%), urinary bladder and mesonephric duct (0.3%), urinary bladder and ureter (0.7%) and kidney (0.07%). However, the predominance of parasites was in the gills, because these were usually ectoparasites.

**Figure 3:**
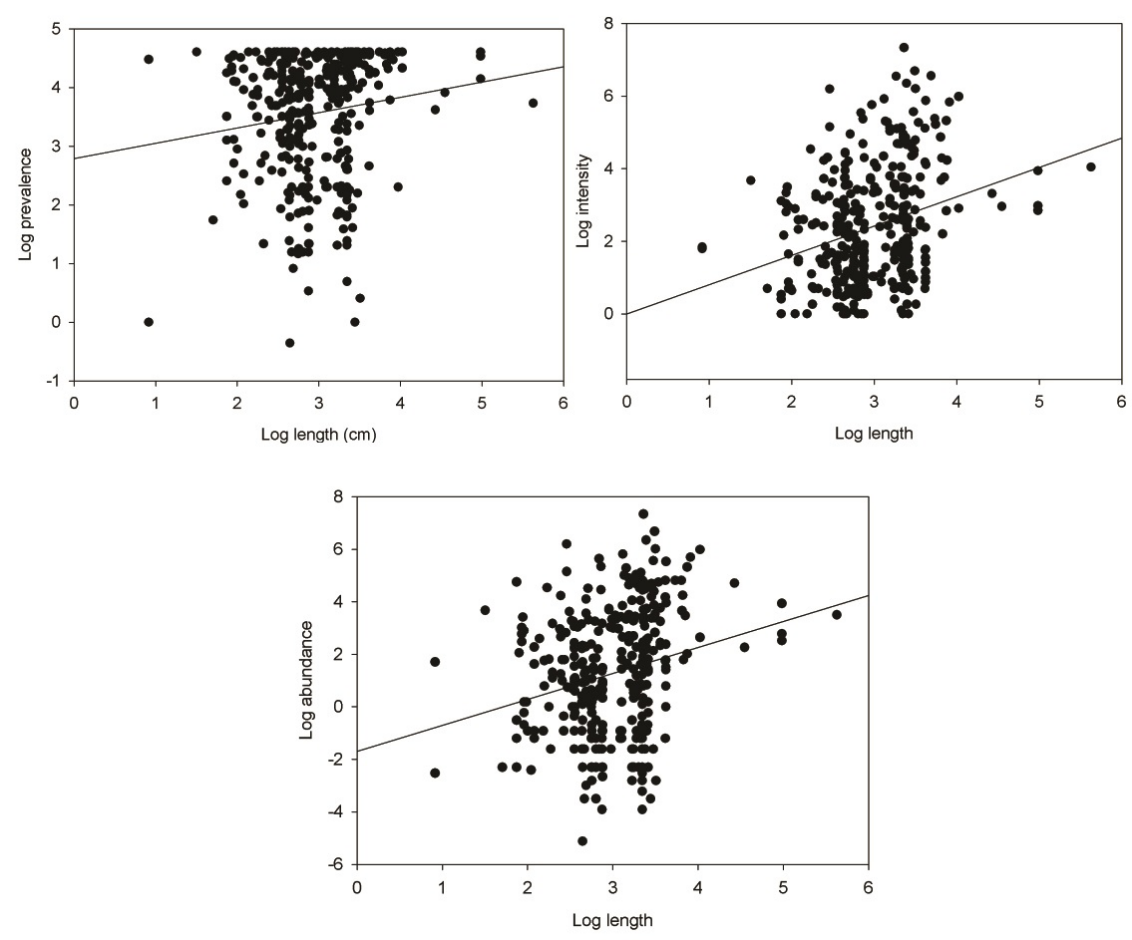
Correlation of host length with prevalence, intensity and abundance of monogeneans for freshwater fish species from Brazil.

A weak positive correlation between the length of hosts and prevalence (*rs* = 0.198, p = 0.001, n = 384), intensity (*rs* = 0.279, p = 0.001, n = 351) and abundance (*rs* = 0.241, p = 0.001, n = 341) of monogeneans was found (Figure 3).

**Figure 4:**
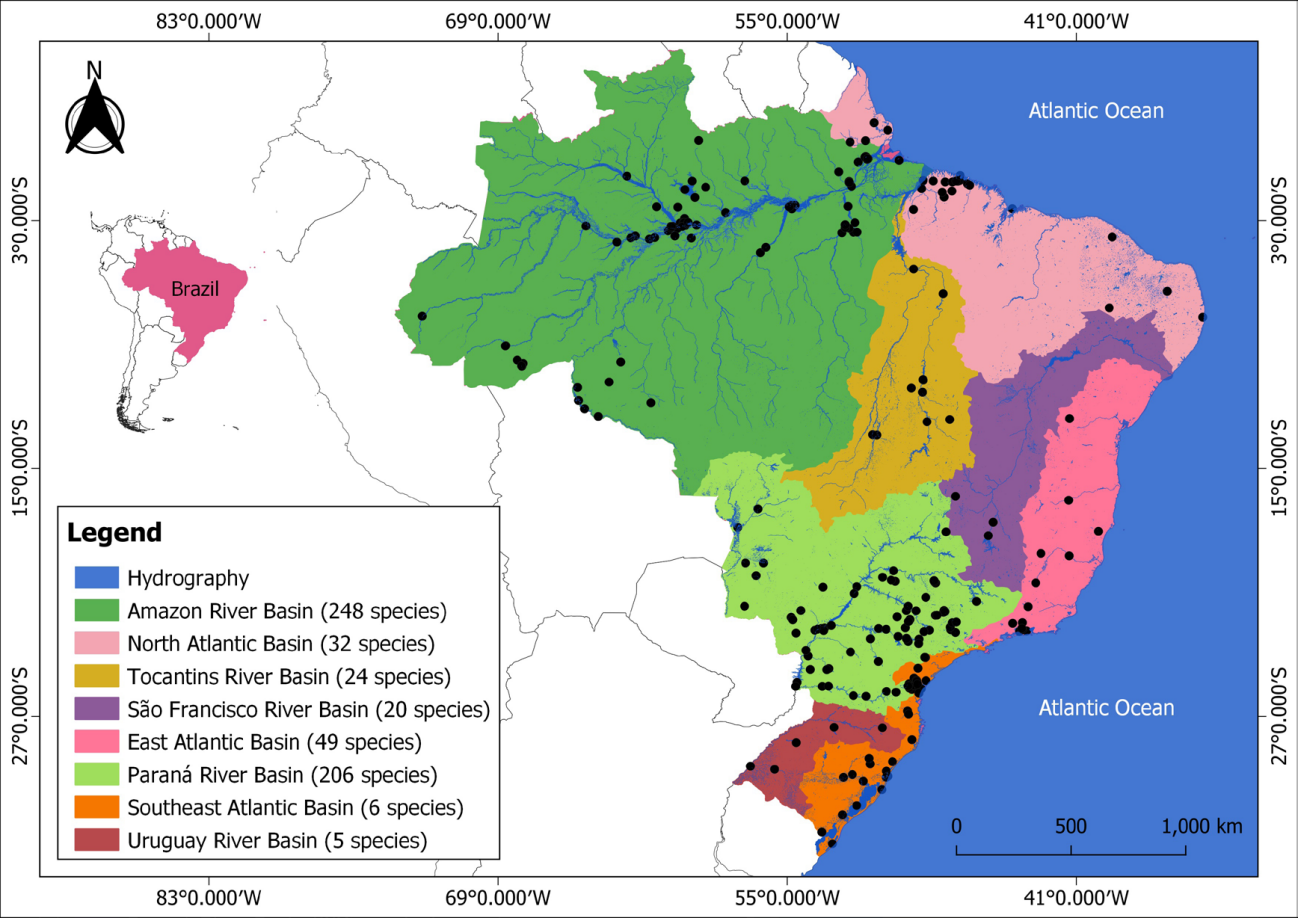
Geographical distribution map of monogenean species in freshwater fish species from Brazil.

Most of the studies and knowledge about species of monogeneans associated to host fish in Brazil are concentrated in the Amazon and Paraná River basins, which present 248 and 206 species describes, respectively (Figure 4).

## Discussion

### Distribution pattern of host-parasite interaction

Monogenean species represent an important component of regional and global biodiversity. Understanding their distribution and which factors drive observed differences in the distribution patterns of these parasites has long been of great interest in parasitology. Estimating the distribution patterns of monogeneans and how they are maintained regarding to their host fish aids to understanding how they diversified, and this information is the cornerstones of parasitic biology and ecology. In this study, we carried out a search for patterns of infection by monogeneans and distribution parameters in 296 freshwater fish of 28 families and 8 orders in host from Brazil, which were mainly species of Characiformes predominantly Characidae (40.9%) Serrasalmidae (8.4%) and Anostomidae (7.4%). In addition, among the host fishes the clade composed by Serrasalmidae popularly known as piranhas (*Serrasalmus* spp. and *Pygocentrus* spp.) stood out in terms of parasite richness of monogenean species per host, and similar a finding was reported by Braga et al. (2014). Characiformes accounts for around 75.0% of freshwater fish in the Neotropical region, with approximately 234 genera and 2,000 species, distributed in different families (Bonato et al., 2017; Froese & Pauly, 2022). Therefore, these results may reflect greater effort on studies on the monogeneans of these taxa of host fish and may also reflect local priorities for parasitological research on these hosts. Despite these studies on the presence of monogeneans in native freshwater fish, the distribution patterns of Brazilian fish in different aquatic ecosystems have been little studied thus far.

The attachment of the monogeneans to a host fish is dependent on both mechanical structures and chemical factors in the parasite (Buchmann & Lindenstrøm, 2002). Analysis of the monogeneans-host interactions is an important way to evaluate the local adaptation of these parasites with heterogeneous distribution in host because infection rates vary between host fish populations. Studies provided evidence that host choice by monogeneans is age-dependent of hosts, and that this life-history trait can play a major role in structuring populations of parasites (Wunderlich et al., 2022). We detected the following patterns within monogenean-host fish interactions in the different basins in Brazil: (a) prevalence ranging from low to high, with abundance and intensity ranging from low to moderate; (b) association with other ectoparasites infracommunities on gills; (c) distribution pattern typically aggregated; (d) occasionally positive correlation of abundance with body size of hosts at the infracommunity level and (e) are usually ectoparasites, infecting mainly gills and tegument. These findings from the present study are in line with other studies, because in most of the parasite-host interaction systems, the gills were the site most frequently infected by monogeneans (Azevedo et al., 2011; Lehun et al., 2020). Monogeneans are selective in their choice of attachment site. This site selection can probably be influenced by factors related to parasites, environment and hosts, and the causative mechanisms may vary between taxa of these parasites. However, some monogeneans preferentially select either gills or tegument. It has been estimated that most of monogeneans are parasites on the tegument or gills of the host fish, and that their mode of life is generally assumed to be ancestral (Cribb et al., 2002; Hoai, 2020; Kuchta et al., 2020). The skin chemical composition of fish is known to be species-specific, with specific proteins (Whittington et al., 2000) and therefore can display some differences between families and fish species. Thus, many taxa of monogeneans have an economic significance due to their parasitic pattern of rapid and unstable population growth, resulting in harm to the hosts, and economic impacts mainly to fish farms (Hoai, 2020).

Different host fish species are exposed to different monogenean species, potentially resulting in different infection rates. In general, the parasite communities are characterized by one or a few numerically dominant species and some rare species. Although this pattern is well recognized, its underlying causes remain unknown (Poulin & Justine, 2008). Parasitism is a wide life strategy among the parasite species of fish; hence, we can reasonably assume that no fish may escape being parasitized. Considering the diversity of fish Brazilian species, we found high diversity of monogeneans associated with their potential hosts that was similar to that reported by Luque et al. (2017). We may suggest that among 238 fish species sampled some, on average, harbor at least one monogenean species whereas others harbor various species of monogeneans. However, the real diversity of these parasites is almost certainly underestimated, often due to the difficulties involved in searching for these extremely small-sized organisms. In the samples of wild fish from Brazil analyzed, we found a moderate to high prevalence of monogenean species, low abundance and intensity ranging from low to moderate, in contrast to farmed fish that had higher abundance and intensity. The prevalence, intensity and abundance of different species in the monogenean communities sampled in this study are clearly unequal. Therefore, we detected variation in infection rates of monogenean species in the host fish that may reflect the fluctuations in environmental conditions (Lacerda et al., 2018; Cavalcanti et al., 2020). The variation in infection patterns of monogeneans could be associated with the great variation in the environmental conditions of the different hydrographic basins and/or with the availability of host fish with varied geographic distribution. In addition, it has been postulated that the rate development of monogeneans can be different between host species influencing generation time, age at sexual maturity as well as size of the adult parasites. The generation time of monogeneans also may differ in different environmental conditions, hosts and localities (Hoai, 2020). A range of dynamic interactions between monogeneans and fish hosts is also responsible for host finding, host specificity and host immunity. Mechanical and chemical factors play a role in these decisive contacts (Buchmann & Lindenstrøm, 2002).

In Brazilian freshwater host fishes, studies focusing the host-specific monogeneans were performed and well discussed by Braga et al. (2014). Host specificity by monogeneans is considered to be a result of various factors including phylogenetic, physiological and ecological aspects. Owing to the close association between host fishes and the monogeneans, evolution of these parasites closely follows the evolutionary history of its hosts (Whittington et al., 2000; Poulin, 2002; Buchmann & Lindenstrøm, 2002; Braga et al., 2014; Mendlová & Šimková, 2014; Řehulková et al., 2021). It has been also suggested that this host specificity improves the reproductive success of the monogenean species, since these parasites only focus on specific organs, and includes a method of coping with the immune system of a single host species or limited range of hosts (Whittington et al., 2000). Monogenean-host interaction are important owing to the close association between hosts and their parasites, and parasite evolution closely can follow the evolutionary history of its host (Braga et al., 2014). However, high host specificity does not necessarily reflect a historical association between hosts and parasites, because host-parasite systems that evolve as a result of host switching may also show a high degree of host specificity (Mendlová & Šimková, 2014). Despite the putative high host specificity of monogeneans, some species had a wide host range that included distantly related hosts because some monogeneans genera were generalists, and this was more evident in the present study than in the findings of Braga et al. (2014). In Congolian cichlids, the absence of strict host-specificity by *Cichlidogyrus* species has been demonstrated, and the occurrence of these monogeneans was not regarded simply as a consequence of their hosts’ distributional range (Vanhove et al., 2013). Řehulková et al. (2021) also demonstrated that *Dactylogyrus marocanus* (El Gharbi & Lambert 1994) has a relatively low level of host-specificity in fish species from the Morocco. This infection of different host taxa can be a strategy for decreasing the chances of local extinction due to fluctuations of host abundance, which may be an adaptive mechanism. In addition, this also may indicate that some fish species with higher parasite richness of monogeneans are sampled more frequently as they exhibit larger distribution ranges or high population densities, such taxa traits representing one of the main determinants of parasite richness reported.

Due to its simple and direct life cycle, morphological adaptation, and a relative host-specificity, gill monogeneans of fish are commonly studied parasites in the context of coevolution and biogeography of host-parasite system (Moreira et al., 2019). Patterns of host distributions of monogeneans in host fish are little known. Monogeneans usually have a free-swimming oncomiracidium that finds and attaches to the hosts. Thus, fish are potentially exposed to essentially the full range of monogenean taxa. The exceptions occur in a few taxa, notably almost all Gyrodactylidae species, where worms transfer directly from host to host (Cribb et al., 2002). Hence, gyrodactylids species are less narrowly specific than other parasitic monogeneans, but this group encompasses both narrowly specific and extremely catholic species (Bakke et al., 2002). In present study, *Gyrodactylus* spp. occurred in 90.0% of Callichthyidae hosts, while the other Siluriformes were infected in most by Dactylogyridae species.

A total of 53 families of monogeneans have been recognized (Cribb et al., 2002); however, Dactylogyridae Bychowsky, 1933 is the most abundant family of monogeneans in freshwater fish (Zago et al., 2020), represented by richest genera and species of these parasites. Gyrodactylidae van Beneden & Hesse, 1863 are ectoparasitic monogeneans that infect mainly teleost fish species. Oviparous gyrodactylids are restricted to South American freshwater catfishes, whereas viviparous gyrodactylids represent one of the most diverse and widespread taxa of monogeneans, with more than 400 species described worldwide. In the Neotropical region, approximately 20 species of *Gyrodactylus* are known (Bueno-Silva & Boeger 2014; Bueno-Silva & Boeger 2019). We also found species of *Gyrodactylus* infecting few species of Characiformes and Cichliformes. There is a total of 402 *Gyrodactylus* species worldwide parasite diverse teleost fishes of 19 orders that infect 45 orders a wide range of fish species: Perciformes>Siluriformes>Cypriniformes>Scorpaeniformes>Characiformes>Anguilliformes (Bakke et al., 2002). Among Neotropical Siluriformes, only species of Callichthyidae, Auchenipteridae, Heptapteridae, Loricariidiae and Pimelodidade are known to be parasitized by gyrodactylids species (Bueno-Silva & Boeger, 2014; Vianna & Boeger, 2019). However, infections by gyrodactylids species are considered accidental in Pimelodidae (Vianna & Boeger, 2019). Nevertheless, the most gyrodactylids species have been described from temperate freshwater fish in Eurasia and North America (Bakke et al., 2002).

Host fish body size has long been considered a determinant of parasite species richness and abundance, although it generally explains only a portion of the interspecific variance in the numbers of parasite species infecting different host species (Sasal & Morand, 1998; Poulin & Leung, 2011). However, the influence of host body size on parasite abundance with is far from universal. Fish body length, in samples of the present study, showed a positive correlation with the prevalence (*rs* =0.198), intensity (*rs* =0.279) and abundance (*rs* =0.241), but these correlation coefficients were low. Sasal & Morand (1998) demonstrated that host size seems to be the most important factor influencing both richness and specificity for monogeneans parasitizing Mediterranean fish species. Poulin & Justine (2008) reported that although the body size variation of host fish on its own has been not account for pronounced differences in abundance among monogenean species within the same community, body size remains an important determinant of abundance as it relates to life history traits underpinning reproductive rates and population growth in the unsaturated communities.

### Geographic distribution

Brazil is considered a hotspot for biodiversity and holds the highest number of freshwater fish species in the Neotropical region. This country has a large number of basins and some of them are long as the Madeira River (3,250 km), São Francisco River (3,160 km), Paraná River (2,940 km), Tocantins River (2,640 km), Uruguay River (1,770 km) and Paraguay River (1,500 km). Also, Brazil has the longest and largest basin in the world, the Amazon River, consisting of the most extensive river system on Earth, occupying a total area of about 6,110,000 km^2^ and represents the largest amount of liquid freshwater on the planet. These basins present differences hydrodynamic related to differenced factors (e.g., size, urban occupation, exploration of electric power and industries, water quality, others) (ANA, 2021). Within this large number of freshwater ecosystems, great fish diversity can be found - about 3,520 fish species (Froese & Pauly, 2022). Many of these fish species are important economically to fishing and aquaculture, for several reasons. Despite the importance of these large basins, the geographic distribution patterns of monogeneans in Brazil are not known, thus far.

Identifying the factors that influence the geographic distribution patterns of parasites is a fundamental challenge in parasite ecology. Understanding which factors drive observed differences in monogenean diversity in Brazilian fishes is of great interest in fish parasitology. At the geographic regional scale, diversity of parasites may be strongly linked to the number of potential host fish species, which in turn is associated to a region’s size, because larger geographical regions supporting a greater number of both host and parasite species (Paterson et al., 2021). Geographical distribution of monogenean species in fish from Brazil may provide insights into the evolution and diversity of parasitic communities. However, the biogeography of these parasites is little known, thus far (Kohn & Cohen, 1998; Cohen & Kohn, 2008), especially because it requires an understanding of host-parasite interactions patterns, information that is scarce as concerns the size of these regions in Brazil. Due to a great heterogeneity of hydrographic basins, Brazil reflects a rich and diversified monogenean fauna. According to Braga et al. (2014), the history of the lineages of each host fish order within Neotropical freshwater seems to have a great influence on the extent of monogenean sharing. The modularity is influenced by both spatial structure and phylogenetic relatedness of species. In addition, that authors reported that in average, 37.0% of modules of networks between host species and monogeneans genera were associated with a particular river basin, and 63.0% of modules were associated with a host family. Hence, spatial structure determines the co-occurrence of host and monogenean species, but their evolutionary history is the main factor defining which interactions are possible.

Historical host–parasite associations between monogeneans and freshwater Neotropical fishes are fascinating due to the host’s geographic isolation in hydrographic basins, which have complex biogeographical histories (Wendt et al., 2022). Parasites are strictly dependent on their hosts for essential resources as nutrients and attachment; thus, their geographical distribution, biodiversity and evolutionary history must, in part, be influenced by the history of their hosts (Reverter et al., 2017; Paterson et al., 2021). For the most Brazilian hydrographic basins there is a lack of knowledge regarding diversity of monogeneans parasitizing host fish species, in contrast to other basins as such Amazon River and Paraná River, which concentrate the most of studies. These differences regarding the distribution and geographic distribution of monogeneans are strongly related to the presence of fish parasitologists in the Amazon region and the Paraná River. Wendt et al. (2022) reported the occurrence of *Characithecium* sp. on the gills of a characid clade containing genera *Astyanax*, *Psalidodon* and *Oligosarcus* distributed across of states from Rio Grande do Sul, Paraná, São Paulo, Bahia, Minas Gerais and Mato Grosso do Sul (Brazil). However, the spatial variation in host-monogenean interactions is one of the major driving forces of this diversity and contributes to the geographic mosaic selection in Brazil. A range of dynamic interactions between monogeneans, fish hosts and environment are responsible for distribution of these parasites for finding their hosts (Buchmann & Lindenstrøm, 2002; Wendt et al., 2022).

There is a great interest in the geographic distribution of monogeneans for regions, which may contribute to global estimates of the distribution of these ectoparasites in host fishes. Hence, global distribution of *Gyrodactylus anguillae* (Ergens 1960) (Hayward et al., 2001a) and distribution of *Gyrodactylus salaris* (Malmberg, 1957) across Europe (Paladini et al., 2021) has been reported. Hayward et al. (2001b) reported the global geographic distribution of *Pseudodactylogyrus anguillae* (Yin & Sproston 1948) Gusev, 1965 and *Pseudodactylogyrus bini* (Kikuchi, 1929) in species of freshwater eels. Studies on the geographic distribution of monogeneans parasitizing Cypriniformes from North America and Europe found that it was affected by phylogeny, indicating an effect of parasite life history on host-specificity. The difference in parasite host range between the two continents could potentially be explained by either the low overall level of sampling activity in North America or an underestimation of parasite diversity in Europe (Kuchta et al., 2020). Studies on the geographic distribution and phylogeography of *Gyrodactylus konovalovi* (Ergens 1976) from Central China suggests their phylogeography was shaped by geological events and climate fluctuations, such as orogenesis, drainage capture changes, and vicariance during the Pleistocene in the Qinling Mountains (Chen et al., 2020). Nevertheless, the global geographic distribution of monogeneans is yet unknown. Our data contribute therefore to studies of elaboration of global maps on the geographic distribution of monogeneans in fish populations.

## Conclusions

The diversity of monogeneans (n = 578) in many host fish taxa of Brazil is certainly underestimated, often due to the difficulties involved in searching for extremely small-sized organisms. At the host species scale, the diversity of monogeneans seems to be linked to a series of host-specific ecological traits (e.g., body size, geographical range, behavior, others), phylogenetic history (e.g., evolutionary age or distinctness), and environmental factors. The results have shown several relevant insights regarding the distribution patterns of monogeneans in host fish across some hydrographic basins of Brazil, and the present study constitutes the most extensive survey regarding species of these parasites in host fish of these basins. Monogeneans have a cosmopolitan distribution and parasitize a diverse range of host fish, and the lack of knowledge regarding diversity of monogeneans parasitizing host fish species in most of hydrographic basins indicate need of enlarged of these studies.

Our limited knowledge on the reproductive strategies of monogeneans in fish species from Brazil is a considerable barrier for protection against diseases and control and treatments. The understanding the life cycle of monogenean species and the influence from environmental factors improves the effectiveness of disease management caused by these parasites. Future research efforts would be best to focus on the fundamental biology of these interesting parasites, also using taxonomic data and infection parameters. These studies will assist in revealing data of intrinsic value to fill up the knowledge gaps and, also, to provide critical suggestions that are more consistent regarding distribution patterns of monogeneans in the host fish of the rich biodiversity of this Neotropical region. Since each fish species from Brazil may have at least two monogenean species, we only know about 6.0% of the species of these parasites. Consequently, many new species yet await discovery and description. So, the potential for expansion of our knowledge about monogeneans is truly in future studies. Monogeneans’ diversity will certainly increase with the increases of broader studies. Furthermore, studies which examine these parasites in all body sites of host fish could overcome the effects of less precise studies of many infection sites. Lastly, all these recommendations may promote further understanding of fish and monogeneans diversity in Brazilian, as well as their interactions; hence, we highlight the future roles both fish biologists and parasitologists may play.
